# Medical students’ relative immunity, or lack thereof, against COVID-19 emotional distress and psychological challenges; a descriptive study from Jordan

**DOI:** 10.12688/f1000research.52051.2

**Published:** 2021-05-24

**Authors:** Khalid Kheirallah, Sarah Bloukh, Wasim Khasawneh, Jomana Alsulaiman, Adi Khassawneh, Abdel-Hameed Al-Mistarehi, Mohammad Alqudah, Lina Elsalem, Samir Al Bashir, Hasan H. Awad, Tariq Al-Shatanawi, Tareq Saleh

**Affiliations:** 1 Department of Public Health, Faculty of Medicine, Jordan University of Science and Technology, Irbid, Jordan; 2 Department of Basic Medical Sciences, Faculty of Medicine at The Hashemite University, Zarqa, Jordan; 3 Department of Pediatrics and Neonatology, Medical School of Jordan University of Science and Technology, Irbid, Jordan; 4 Department of Pediatrics, Faculty of Medicine at Yarmouk University, Irbid, Jordan; 5 Department of Pathology and Microbiology, Medical School of Jordan University of Science and Technology, Irbid, Jordan; 6 Department of Pharmacology, Medical School of Jordan University of Science and Technology, Irbid, Jordan; 7 Department of Public Health, Faculty of Medicine at Al-Balqa Applied University, Al-Salt, Jordan

**Keywords:** COVID-19, emotional distress, emotional changes, medical students, Jordan, social media, medical school, SARS-CoV-2.

## Abstract

**Background:** Emotional distress is a major impact of COVID-19 among not only the general public but also healthcare workers including medical students. This study aimed at describing self-reported changes in emotional reactions associated with COVID-19 among medical students in Jordan and to assessing the potential effect of social media utilization on emotional distress among this group.

**Methods:** A cross-sectional design was utilized to collect data early on during the outbreak in Jordan. All medical students in Jordan were eligible to complete an online questionnaire assessing self-reported emotional reactions to COVID-19 that covered four main domains: negative emotion (anxiety, worry, depression, panic, loneliness, and nervousness), positive emotion (happiness, joy, and excitement), sleep disorders (insomnia, shallow sleep, nightmares, and insufficient sleep), and aggression (verbal argument and physical fighting). The frequency of social media utilization as a main source of COVID-19 information was also assessed.

**Results:** 59.9% of participants were females, 64.9% were enrolled at the two major medical schools in Jordan, and 59.6% were in the pre-clinical stage (years). A significant proportion of participants self-reported increased negative emotional levels of anxiety (49.2%), worry (72.4%), depression (23.1%), panic (22.6%), and nervousness (38.2%) and decreased positive emotional levels of happiness (44.8%), joy (47.3%), and feelings of excitement (45.1%). Self-reported sleep disorders were not as common (less than 15% for any of the four items), while arguing with others was at 26.7%. Significant differences by gender and academic year were detected. Almost half of participants reported using social media as a main source of COVID-19 information “most/all-the-times” with a significant effect of such on reducing emotional distress.

**Conclusion:** The results suggest a potential effect of COVID-19 on the emotional distress of medical students. Addressing and mitigating such effects is crucial. The potential buffering effect of social media should be further investigated.

## Introduction

COVID-19 is shadowing its effect on almost all individuals living today. The health, safety, and well-being of individuals and communities are all expected to be affected. These effects may include anxiety, fear, frustration, loneliness, anger, boredom, depression, stress, and avoidance behaviour ( Talevi *et al.*, 2020) and may simply translate into emotional disturbances not only among those with the disease but also the general population and other sub-groups ( Pfefferbaum & North, 2020).

Certain population subgroups may be more vulnerable than others to the psychosocial effects of COVID-19. Healthcare workers, including medical students, especially in resource-limited countries, are particularly vulnerable to emotional distress during COVID-19 given their high risk of exposure to the infection, potential shortage of personal protective equipment, longer work hours, and involvement in emotionally and ethically fraught resource-allocation decisions ( Greenberg, 2020; Loch *et al.*, 2020; Samrah, Al-Mistarehi, Aleshawi, *et al.*, 2020; Talevi *et al.*, 2020; Vindegaard & Eriksen Benros, 2020).

Studies have reported that medical schools have one of the most demanding academic programs ( Wolf, 1994) and include students at higher risk for developing anxiety disorders, compared to the general population, even under normal circumstances ( Quek *et al.*, 2019). Medical emergencies destabilize medical students’ already vulnerable psychological status leading to unfavourable effects on their learning journey ( Al-Rabiaah *et al.*, 2020), physical and mental health, and their professional identity formation ( Chandratre, 2020). While medical students may experience increasing level of anxiety and stress due to the COVID-19 disruption ( Lasheras *et al.*, 2020; Ullah & Amin, 2020), they are the least likely to seek support for mental health problems ( Chandratre, 2020).

During outbreaks, medical students are expected to become part of the frontline workforce ( Kinder & Harvey, 2020; O’Byrne *et al.*, 2020), which may further expose them to psychological distress. Therefore, it is important to assess and safeguard medical students’ mental health status and to implement proper strategies to support their mental well-being. Providing a clear understanding of the extent of psychological impact of COVID-19 infection should, then, be clearly established.

Jordan is a developing country in the Middle East region and reported its first case of SARS-CoV-2 infection in early March, 2020 ( Samrah, Al-Mistarehi, Ibnian, *et al.*, 2020). A series of *strict* non-pharmaceutical intervention (NPI) measures were then implemented ( Kheirallah *et al*., 2020; Samrah, Al-Mistarehi, Aleshawi, *et al*., 2020) that included a curfew and a total shutdown. By mid-March, 2020, all educational institutions, including medical schools, were ordered closed and online education was, suddenly, the only option ( Sindiani *et al*., 2020). This may have increased the level of psychological distress among medical students ( Elsalem *et al*., 2020). The current study described the self-reported changes in emotional reactions associated with COVID-19 among medical students in Jordan and investigated the potential effect of utilizing social media as a main source of COVID-19 information on these emotional changes.

## Methods

A descriptive cross-sectional design collected data on medical students between March 17 and 20, 2020, shortly after Jordan initiated the NPI measures stated earlier. Data were collected from all medical schools (N = 6) in Jordan and included all years (year 1 to year 6). The 6-year program consists of two stages: preclinical, the first three years of the program, and clinical, the last three years. *The minimum sample size required to detect a 10% difference in any of the emotional items under investigations was estimated at 600 students (alpha = 0.05, power = 0.8).* Considering a response rate of around 20% for online questionnaires among the sample population ( Khasawneh *et al*., 2020), an online data collection tool, using Google forms, was utilized and a link was emailed to a randomly selected sample (N = 3,000 out of the total estimated number of medical students in Jordan) that was proportionate to the year of study and the medical school.

Participants’ emotional reactions to COVID-19 pandemic were self-reported using 15 items covering four main domains: negative emotions (anxiety, worry, depression, panic, loneliness, and nervousness), positive emotions (happiness, joy, and excitement), sleep disorders (insomnia, shallow sleep, nightmares, and insufficient sleep), and aggression (verbal argument and physical fighting). Medical students compared the frequency of each item after the onset of the pandemic with before the pandemic using a three-point scale (from “1 = less compared to the days before the pandemic” to “3 = more compared to the days before the pandemic”). For selected items, responses were randomized. Participants were also asked to assess their usage of social media as a main source of information for COVID-19 using a three-point Likert scale (from *“1 = never” to “3 = most/all the time”*). The questionnaire was reviewed by expert panel before being piloted on 20 students.

The Institutional Review Boards of The Hashemite University (5/2/2019/2020) and Al-Balqa Applied University (26/3/1/804) approved this study. An online consent form was obtained by all participants prior to being directed to take the questionnaire.

Each emotional item was reported overall and by gender, academic level and by social media utilization using numbers and percentages. A Chi-squared test was used to compare percentages for each emotional item. The Alpha level was set at 0.05.

## Results

A total of 1,404 students participated in the current study (response rate = 46.8%). The distribution of participants by gender, university, and year is presented in Table 1. About two-thirds (59.9%) of participants were females, 64.9% were enrolled at the two well-established medical schools at the University of Jordan (39.1%) and Jordan University of Science and Technology (JUST) (25.8%), and 59.6% were in the pre-clinical stage of their medical education.

**Table 1.** Study participants’ background characteristics.

		Number	*Percent*
Gender			
	Female	836	*59.5*
	Male	568	*40.5*
	**Total**	**1,404**	***100.0***
University			
	Al-Balqa`	117	*8.4*
	Hashemite	192	*13.7*
	Jordan	550	*39.1*
	JUST	362	*25.8*
	Mutah	62	*4.4*
	Yarmouk	121	*8.6*
	**Total**	**1,404**	***100.0***
School Year			
Pre-clinical	1 ^st^ year	145	*10.3*
	2 ^nd^ year	348	*24.8*
	3 ^rd^ year	343	*24.5*
Clinical	4 ^th^ year	264	*18.8*
	5 ^th^ year	176	*12.5*
	6 ^th^ year	128	*9.1*
	**Total**	**1,404**	***100.0***

Overall, almost half of the participants (49.2%) self-reported that they experienced increased levels of anxiety. This was significantly more prevalent among females (58.6%) than males (35.5%) (p<0.0001). Self-reported worry, which was the most commonly reported negative emotion experienced among all participants (72.4%), was significantly more prevalent among females than males (79.5% *vs* 61.9%, p<0.0001). Similarly, self-reported depression and panic, respectively, were more prevalent among females (26.8% and 28.9%) compared with males (17.8% and 13.4%) (p-value for both comparisons <0.0001) ( Table 2).

**Table 2.** Distribution of study participants by study items and by gender and academic study level (N = 1,404).

			Total (N = 1,404)		Female (N = 835)		Male (N = 569)		*P-Value*	Pre-clinical (N = 837)		Clinical (N = 567)		*P-Value*
	Item	Response	n	*%*	n	*%*	n	*%*		n	*%*	n	*%*	
Negative emotions	Anxiety	No	713	*50.8%*	346	*41.4%*	367	*64.5%*	*0.000*	414	*49.5%*	299	*52.7%*	*0.232*
		Yes Increased	691	*49.2%*	489	*58.6%*	202	*35.5%*		423	*50.5%*	268	*47.3%*	
	Worry	No	387	*27.6%*	170	*20.4%*	217	*38.1%*	*0.000*	218	*26.0%*	169	*29.8%*	*0.128*
		Yes Increased	1,017	*72.4%*	665	*79.6%*	352	*61.9%*		619	*74.0%*	398	*70.2%*	
	Depression	No	1,079	*76.9%*	611	*73.2%*	468	*82.2%*	*0.000*	620	*74.1%*	459	*81.0%*	*0.003*
		Yes Increased	325	*23.1%*	224	*26.8%*	101	*17.8%*		217	*25.9%*	108	*19.0%*	
	Panic	No	1,087	*77.4%*	594	*71.1%*	493	*86.6%*	*0.000*	613	*73.2%*	474	*83.6%*	*0.000*
		Yes Increased	317	*22.6%*	241	*28.9%*	76	*13.4%*		224	*26.8%*	93	*16.4%*	
	Loneliness	No	909	*64.7%*	565	*67.7%*	344	*60.5%*	*0.006*	522	*62.4%*	387	*68.3%*	*0.026*
		Yes Increased	495	*35.3%*	270	*32.3%*	225	*39.5%*		315	*37.6%*	180	*31.7%*	
	Nervousness	No	868	*61.8%*	452	*54.1%*	416	*73.1%*	*0.000*	481	*57.5%*	387	*68.3%*	*0.000*
		Yes Increased	536	*38.2%*	383	*45.9%*	153	*26.9%*		356	*42.5%*	180	*31.7%*	
Sleep disorders	Insomnia	No	1,231	*87.7%*	720	*86.2%*	511	*89.8%*	*0.047*	715	*85.4%*	516	*91.0%*	*0.002*
		Yes Increased	173	*12.3%*	115	*13.8%*	58	*10.2%*		122	*14.6%*	51	*9.0%*	
	Shallow sleep	No	1,222	*87.0%*	712	*85.3%*	510	*89.6%*	*0.019*	715	*85.4%*	507	*89.4%*	*0.029*
		Yes Increased	182	*13.0%*	123	*14.7%*	59	*10.4%*		122	*14.6%*	60	*10.6%*	
	Nightmares	No	1,256	*89.5%*	726	*86.9%*	530	*93.1%*	*0.000*	739	*88.3%*	517	*91.2%*	*0.092*
		Yes Increased	148	*10.5%*	109	*13.1%*	39	*6.9%*		98	*11.7%*	50	*8.8%*	
	Insufficient sleep	No	1,230	*87.6%*	717	*85.9%*	513	*90.2%*	*0.017*	717	*85.7%*	513	*90.5%*	*0.008*
		Yes Increased	174	*12.4%*	118	*14.1%*	56	*9.8%*		120	*14.3%*	54	*9.5%*	
Aggression	Argue with others	No	1,029	*73.3%*	610	*73.1%*	419	*73.6%*	*0.854*	587	*70.1%*	442	*78.0%*	*0.001*
		Yes Increased	375	*26.7%*	225	*26.9%*	150	*26.4%*		250	*29.9%*	125	*22.0%*	
	Physical fight	No	1,333	*94.9%*	787	*94.3%*	546	*96.0%*	*0.173*	778	*93.0%*	555	*97.9%*	*0.000*
		Yes Increased	71	*5.1%*	48	*5.7%*	23	*4.0%*		59	*7.0%*	12	*2.1%*	
Positive emotions	Happiness	No	775	*55.2%*	445	*53.3%*	330	*58.0%*	*0.090*	457	*54.6%*	318	*56.1%*	*0.585*
		Yes Decreased	629	*44.8%*	390	*46.7%*	239	*42.0%*		380	*45.4%*	249	*43.9%*	
	Joy	No	740	*52.7%*	416	*49.8%*	324	*56.9%*	*0.009*	433	*51.7%*	307	*54.1%*	*0.384*
		Yes Decreased	664	*47.3%*	419	*50.2%*	245	*43.1%*		404	*48.3%*	260	*45.9%*	
	Feeling excitement	No	771	*54.9%*	418	*50.1%*	353	*62.0%*	*0.000*	447	*53.4%*	324	*57.1%*	*0.172*
		Yes Decreased	633	*45.1%*	417	*49.9%*	216	*38.0%*		390	*46.6%*	243	*42.9%*	

About 35% and 38% of surveyed students self-reported increased levels of loneliness and nervousness, respectively. While significantly more males (39.5%) than females reported increased level of loneliness (p=0.006), more females (45.9%) than males (26.9%) self-reported increased nervousness levels (p<0.001).

On the other hand, self-reported anxiety and worry were not significantly different between students in the pre-clinical *vs* clinical years while depression and panic, respectively, were significantly higher among students in pre-clinical years (25.9% and 26.8%) compared with their counterparts in the clinical years (19.0% and 16.4%) (p=0.003 and <0.001). Similarly, self-reported loneliness and nervousness, respectively, were significantly more common among students in the pre-clinical years (37.6% and 42.5%) compared to their counterparts in the clinical years (31.7% and 31.3%) (p=0.026 and <0.001).

About 13% of students self-reported experiencing increased insomnia, shallow sleep, nightmares, or insufficient sleep. In general, females self-reported experiencing significantly more sleeping problems than males. Likewise, students in the pre-clinical years experienced sleep problems (insomnia, shallow sleep, and insufficient sleep) significantly more frequently than those in their clinical years.

While about one-quarter of participants (26.7%) self-reported increased level of arguing with others, 5.1% self-reported increasing physical fight. However significant differences in aggression variables were not detected by gender, students in the pre-clinical years self-reported higher levels of both arguing with others (26.9%), compared with their counterparts in the clinical years (22.0%) (p=0.001), and physical fights (7.0%), compared with clinical years students (2.1%) (p<0.001).

Approximately half of the participants self-reported a decrease in their level of positive emotions, namely happiness (44.8%), joy (47.8%), and excitement (45.1%). Self-reported decrease in the levels of joy and excitement, respectively, were statistically more prevalent among females (50.2% and 49.9%) compared with males (43.1% and 38.0%) (p=0.009 and <0.001). Significant differences in each of the three positive emotions by academic levels were not statistically significant.

Overall, 37.9% of study participants reported never using social media as a source of information for COVID-19, while almost half (45.6%) reported using it “most/all the times” ( Table 3). Statistically significant relationships were detected between social media as a source for COVID-19 information and anxiety (p=0.002), worry (0.016), panic (<0.001), loneliness (0.003), and nervousness (0.004). Among those who reported to use social media most/all the times as a source of information about COVID-19, the prevalence estimates of self-reported anxiety, worry, panic, loneliness, and nervousness, respectively, were less than that among their counterparts who never used it (47.3% *vs* 55.6%, 70.9% *vs* 77.8%, 20.3% *vs* 28.9%, 31.7% *vs* 41.5%, and 36.1% *vs* 44.2%). Changes in sleep disorder and aggression variables, on the other hand, were not significantly different by social media.

**Table 3.** Distribution of study participants by social media use as a main source of COVID-19 information and by items under investigation

				Use of social media as a main source of COVID-19 Information						
Item				Never (N=532)		Rarely/sometimes (N=232)		Most/All the times (N=640)		*P-value*
				Number	*Percent*	Number	*Percent*	Number	*Percent*	
	Overall			532	*37.9%*	232	*16.5%*	640	*45.6%*	
Negative emotion	Anxiety	No		241	*46.1%*	135	*58.2%*	337	*52.7%*	*0.002*
		Yes Increased		291	*55.6%*	97	*41.8%*	303	*47.3%*	
	Worry	No		125	*23.9%*	76	*32.8%*	186	*29.1%*	*0.016*
		Yes Increased		407	*77.8%*	156	*67.2%*	454	*70.9%*	
	Depression	No		405	*77.4%*	181	*78.0%*	493	*77.0%*	*0.841*
		Yes Increased		127	*24.3%*	51	*22.0%*	147	*23.0%*	
	Panic	No		381	*72.8%*	196	*84.5%*	510	*79.7%*	*0.000*
		Yes Increased		151	*28.9%*	36	*15.5%*	130	*20.3%*	
	Loneliness	No		315	*60.2%*	157	*67.7%*	437	*68.3%*	*0.003*
		Yes Increased		217	*41.5%*	75	*32.3%*	203	*31.7%*	
	Nervousness	No		301	*57.6%*	158	*68.1%*	409	*63.9%*	*0.004*
		Yes Increased		231	*44.2%*	74	*31.9%*	231	*36.1%*	
Sleep disorders	Insomnia	No		456	*87.2%*	207	*89.2%*	568	*88.8%*	*0.213*
		Yes Increased		76	*14.5%*	25	*10.8%*	72	*11.3%*	
	Shallow sleep	No		454	*86.8%*	211	*90.9%*	557	*87.0%*	*0.105*
		Yes Increased		78	*14.9%*	21	*9.1%*	83	*13.0%*	
	Nightmares	No		469	*89.7%*	207	*89.2%*	580	*90.6%*	*0.388*
		Yes Increased		63	*12.0%*	25	*10.8%*	60	*9.4%*	
	Insufficient sleep	No		466	*89.1%*	200	*86.2%*	564	*88.1%*	*0.749*
		Yes Increased		66	*12.6%*	32	*13.8%*	76	*11.9%*	
Aggression	Argue with others	No		388	*74.2%*	173	*74.6%*	468	*73.1%*	*0.888*
		Yes Increased		144	*27.5%*	59	*25.4%*	172	*26.9%*	
	Physical fight	No		500	*95.6%*	225	*97.0%*	608	*95.0%*	*0.220*
		Yes Increased		32	*6.1%*	7	*3.0%*	32	*5.0%*	
Positive emotion	Happiness	No		298	*57.0%*	128	*55.2%*	349	*54.5%*	*0.879*
		Yes Decreased		234	*44.7%*	104	*44.8%*	291	*45.5%*	
	Joy	No		272	*52.0%*	124	*53.4%*	344	*53.8%*	*0.650*
		Yes Decreased		260	*49.7%*	108	*46.6%*	296	*46.3%*	
	Feeling excitement	No		277	*53.0%*	138	*59.5%*	356	*55.6%*	*0.148*
		Yes Decreased		255	*48.8%*	94	*40.5%*	284	*44.4%*	

## Discussion

The current descriptive study assessed the self-reported emotional changes following the COVID-19 pandemic among medical students in Jordan. Participants were found to have increased levels of almost all self-reported negative emotions and decreased levels of almost all positive emotions. These changes were more prevalently the case with females and preclinical students. Utilizing social media as a main source of COVID-19 information should be further investigated as having a potential “buffering effect” against emotional changes under investigation.

Our results suggest that medical students may not be immune against COVID-19 emotional distress and increased psychological challenges. This could potentially usher a period of adjustment and may produce significant mental health issues. Addressing and mitigating the negative effects of public health emergencies on the mental health of medical students seem to be critical.

Overall, preclinical level students seemed to have a greater increase in almost all negative emotions compared with clinical students. The reason why preclinical students might have had a greater increase in negative emotions could be attributed to their lesser experience in the clinical field and lesser understanding of the pandemic in general. However, it is interesting to note that the two negative emotions that showed no significant difference between preclinical and clinical students were worry and anxiety. When considering that medical students are already exposed to high levels of anxiety disorders ( Quek *et al.*, 2019), higher levels of anxiety are than expected. Exacerbation of a pre-existing emotional distress among medical students due to COVID-19 were previously suggested ( Ullah & Amin, 2020).

Gender differences observed in the current study suggest vulnerability of female medical students to emotional distress more than male students. Females are generally more susceptible to emotional changes due to the hormonal fluctuations that are part of their physiology. It can be expected, thus, that several chemical mediators have a potential of elevating emotional distress in females. Beyond biological considerations, the conservative gender roles present different expectations of behaviours based on gender. Previous research have emphasized the gender roles and traits (masculinity in particular) and explained part of the gender differences in emotional distress and coping mechanisms ( Mayor, 2015). On the other hand, gender differences in emotional intelligence, test stress, coping and academic stress were also suggested to contribute to similar observations ( Alzahem *et al*., 2011; Babar *et al*., 2015; Elsalem *et al*., 2020). Our findings that female medical students self-reported higher rates of depression, anxiety, worry, nervousness, and panic, while male students reported higher rates of loneliness, are in line with the literature addressing such gender roles.

In Jordan, about 7% of medical students reported that they became obsessed with precautionary measures related to COVID-19 ( Khasawneh *et al*., 2020), while about half self-reported distance education as a main source of worry and stress. Such negative emotions were attributed to the learning and assessment models used during the pandemic as students were not familiar with distance learning ( Elsalem *et al*., 2020). The difference in prevalence estimates of distress in different studies may be attributed to several factors among which are the length of quarantine period, environment, contact with COVID-19 patients, and different coping styles of individuals.

Medical students who self-reported utilizing social media as a source of COVID-19 information more frequently reported lower levels of emotional distress compared to those who never utilized it for such. The role that social media can play in risk perception and dissemination of reliable information during pandemics could be critical ( Albarrak *et al.*, 2019). While the information available about the pandemic may be a concern for COVID-19 infodemic among the general public, medical students seem to have utilized social media as a mitigation source to better understand the disease dynamics. This could have been reflected on the lower level of emotional distress reported among students who used social media more often. Medical students, therefore, by accessing social media, may be better equipped to avoid misinformation and to distinguish rumours from reality ( Karasneh *et al*., 2020). Still, our results contradict reports where young people tend to obtain a large amount of information from social media which can easily be a trigger for stress and anxiety ( Qiu *et al*., 2020). Assessment of the role of social media on medical students’ emotional distress should be further investigated using both qualitative and quantitative methods.

It is important to note that, in the current study, all estimates were self-reported and that we did not use standardized tools. This could have called out for over-estimation of the prevalence rates and over presentation of reported emotional changes. The cross-sectional nature of the study limits temporality. It will be imperative for other research groups to include longitudinal aspects in their study design and to use standardized screening tools as well as clinical assessment among this vulnerable group.

## Conclusion

Our results support the notion to screen for mental health problems among medical students and to invest in mental health infrastructures. Psychoeducation, and psychosocial support should be seriously considered within health education programs at medical schools and should be fine-tuned by gender. The role of social media within the context of a classical medical educational system should be further investigated and utilized as a mediating factor towards better mental health and psychosocial support.

## Underlying data

In compliance with IRB guidelines specified for this research activity, data will be shared by the corresponding author following official request for research with clear objectives that is initially approved by the corresponding author.
