# Supplementary material for: Medical students’ relative immunity, or lack thereof, against COVID-19 emotional distress and psychological challenges; a descriptive study from Jordan
Source: F1000Res. 2021 May 24;10:297. Originally published 2021 Apr 19. [Version 2] doi: 10.12688/f1000research.52051.2 (PMC8108551; doi:10.12688/f1000research.52051.2)
Supplement: Peer review history

# Peer review: Reviewer response for version 2

**DOI:** 10.5256/f1000research.56594.r85935

**Published:** 2021-06-15

**Authors:** Tariq Kewan

**Affiliations:** 1 Department of Internal Medicine, Cleveland Clinic Fairview Hospital, Cleveland, OH, USA; 2 Translational Hematology and Oncology, Cleveland Clinic, Cleveland, OH, USA

No further comments.

Is the work clearly and accurately presented and does it cite the current literature?

Yes

If applicable, is the statistical analysis and its interpretation appropriate?

Yes

Are all the source data underlying the results available to ensure full reproducibility?

Yes

Is the study design appropriate and is the work technically sound?

Partly

Are the conclusions drawn adequately supported by the results?

No

Are sufficient details of methods and analysis provided to allow replication by others?

No

Reviewer Expertise:

Oncology, COVID-19 treatment, Coagulopathy, Acute leukemia, blood disorders, critical care

I confirm that I have read this submission and believe that I have an appropriate level of expertise to confirm that it is of an acceptable scientific standard.

# Peer review: Reviewer response for version 2

**DOI:** 10.5256/f1000research.56594.r85933

**Published:** 2021-06-01

**Authors:** Nedal Alnawaiseh

**Affiliations:** 1 Department of Public Health, School of Medicine, University of Mutah, Al-Karak, Jordan

I approve this version.

Is the work clearly and accurately presented and does it cite the current literature?

Yes

If applicable, is the statistical analysis and its interpretation appropriate?

Yes

Are all the source data underlying the results available to ensure full reproducibility?

Yes

Is the study design appropriate and is the work technically sound?

Yes

Are the conclusions drawn adequately supported by the results?

Yes

Are sufficient details of methods and analysis provided to allow replication by others?

Yes

Reviewer Expertise:

Epidemiologist, Biostatician, Public health and research expertise.

I confirm that I have read this submission and believe that I have an appropriate level of expertise to confirm that it is of an acceptable scientific standard.

# Peer review: Reviewer response for version 1

**DOI:** 10.5256/f1000research.55277.r83592

**Published:** 2021-05-07

**Authors:** Walid Alali

**Affiliations:** 1 Department of Epidemiology and Biostatistics, Faculty of Public Health, Kuwait University, Safat, Kuwait

The manuscript present descriptive findings from a cross-sectional study conducted via a questionnaire tool to assess emotional distress during COVID-19 pandemic on medical students in Jordan. There were reports of significant increase in negative emotional mental health issues. The findings add to the scientific literature on mental health issues related to COVID-19 pandemic restrictions in a specific group of people.

 My minor comments:

1. The probability sampling plan is not clear as authors mentioned "students were selected randomly". Needs explanation.
2. The questionnaire tool should be included as an appendix.
3. Please add a statistical/data analysis statement.

Is the work clearly and accurately presented and does it cite the current literature?

Yes

If applicable, is the statistical analysis and its interpretation appropriate?

Partly

Are all the source data underlying the results available to ensure full reproducibility?

Yes

Is the study design appropriate and is the work technically sound?

Yes

Are the conclusions drawn adequately supported by the results?

Yes

Are sufficient details of methods and analysis provided to allow replication by others?

Yes

Reviewer Expertise:

Epidemiology; Infectious Diseases; Public Health; Antimicrobial Resistance.

I confirm that I have read this submission and believe that I have an appropriate level of expertise to confirm that it is of an acceptable scientific standard.

# Peer review: Reviewer response for version 1

**DOI:** 10.5256/f1000research.55277.r83591

**Published:** 2021-04-29

**Authors:** Tariq Kewan

**Affiliations:** 1 Department of Internal Medicine, Cleveland Clinic Fairview Hospital, Cleveland, OH, USA; 2 Translational Hematology and Oncology, Cleveland Clinic, Cleveland, OH, USA

In this article Kheirallah and colleagues studied the impact of COVID-19 pandemic on the medical student emotional distress using a self reported questionnaire. In addition Authors analyzed the impacts of social media utilization as a source of information on the the emotional factors, below are some comments to consider: 

1. The authors used a self reported cross sectional design to analyze the outcomes, however targeted population size and response rates were not reported. It is also worth to mention the response rate by each different university.
2. Can the authors explain why they considered response rate of 20% to be sufficient, please cite a paper. 
3. How the authors guaranteed that each participant will respond one time only?
4. Can you provide the time period of the study?
5. Authors need to provide the questionnaire used in the study to be published as a supplementary with the article. 
6. The definition of different symptoms reported was not specified. For example what is the difference between anxiety and panic or worry and anxiety? This also will raise the question regarding participant understanding of the questions. Did the authors provide explanations for the participant to ensure understanding. Can the authors provide specific definition for each symptoms / disorder they reported, for example what is the dentition of insomnia?
7. Results are conflicting. In table 2 , 72.2% of all participant reported worry but at the same time 44.8% reported increased happiness. This will raise the concern of appropriate understanding of the participants. 
8. Authors concluded that social media use may have a buffering effect on emotional distress which is wrong. This conclusion cant be made based on the study design provided. Authors just described the differences in the prevalence of different symptoms / diseases between three different groups based on the use of social media as a source of information. Other variables / confounders were not taken into consideration and this conclusion is not appropriate. 

Is the work clearly and accurately presented and does it cite the current literature?

Yes

If applicable, is the statistical analysis and its interpretation appropriate?

Yes

Are all the source data underlying the results available to ensure full reproducibility?

Yes

Is the study design appropriate and is the work technically sound?

Partly

Are the conclusions drawn adequately supported by the results?

No

Are sufficient details of methods and analysis provided to allow replication by others?

No

Reviewer Expertise:

Oncology, COVID-19 treatment, Coagulopathy, Acute leukemia, blood disorders, critical care

I confirm that I have read this submission and believe that I have an appropriate level of expertise to confirm that it is of an acceptable scientific standard, however I have significant reservations, as outlined above.

# Response

**Published:** 2021-04-29

**Authors:** khalid kheirallah

**Affiliations:** Jordan University of Science and Technology, Jordan

**We appreciate your opinion and feedback. Kindly note our response in line with the provided comments.**

 In this article Kheirallah and colleagues studied the impact of COVID-19 pandemic on the medical student emotional distress using a self reported questionnaire. In addition Authors analyzed the impacts of social media utilization as a source of information on the the emotional factors, below are some comments to consider: 

1. The authors used a self reported cross sectional design to analyze the outcomes, however targeted population size and response rates were not reported. It is also worth to mention the response rate by each different university.

- **Thank you for this comment. We have updated the manuscript to state that the total population of medical students is 10,000. Out of the sample selected for this survey, a total of 3,000 as already stated, the response rate was added to the results section (46.8%). **

 

1. Can the authors explain why they considered response rate of 20% to be sufficient, please cite a paper. 

- **Thank you for this comment. We have a previous experience with this population and a response rate was less than 30%. Being conservative, we have decided that a 20% is suitable. A reference is already inserted for which we had a 20% response rate. **

1. How the authors guaranteed that each participant will respond one time only?

- **Thank you for this valuable note. We have no guarantee that each student will fill the questionnaire only once. But we have no reason to believe that medical students will fill more than one time. **

1. Can you provide the time period of the study?

- **Thank you for this comment. The study stated, under methods, that this survey was conducted between March 17 and 20, 2020. **

1. Authors need to provide the questionnaire used in the study to be published as a supplementary with the article. 

- **Thank you for this note. We have now updated the supplementary materials to include the questionnaire. **

1. The definition of different symptoms reported was not specified. For example what is the difference between anxiety and panic or worry and anxiety? This also will raise the question regarding participant understanding of the questions. Did the authors provide explanations for the participant to ensure understanding. Can the authors provide specific definition for each symptoms / disorder they reported, for example what is the dentition of insomnia?

- **Thank you for this note. We have defined each term as self-reported. It was meant to screen and prioritize potential mental health issues using self-reported responses. This was stated as part of the limitations. **

1. Results are conflicting. In table 2 , 72.2% of all participant reported worry but at the same time 44.8% reported increased happiness. This will raise the concern of appropriate understanding of the participants. 

- **Thank you for this note. The potential overlap is possible given the way the study was conducted. It may be true that students are worried about their educational process at the early stages of the pandemic, still feel happy being able to spend more time with their families. This issue needs further investigation using qualitative study design as positive and negative emotions are possible given the circumstances of uncertainties. **
- **Qualitative investigations of the study results was suggested and recommended as part of the study’s future directions in multiple locations.**

1. Authors concluded that social media use may have a buffering effect on emotional distress which is wrong. This conclusion cant be made based on the study design provided. Authors just described the differences in the prevalence of different symptoms / diseases between three different groups based on the use of social media as a source of information. Other variables / confounders were not taken into consideration and this conclusion is not appropriate. 

- **Thank you for this note. Social media utilization has been suggested to “moderate” the relationship between multiple variables and mental health issues. As such, our results of potential differences in mental health variables by social media utilization *may *suggest a buffering effect. Future studies, including follow-up designs are then needed to investigate this relationship. This is what was suggested in the conclusion of the study. As such, the effect of social media utilization may, or may not, be a factor in the buffering suggested. The conclusion, as such, has been worded to reflect this. Edits were made. **

# Peer review: Reviewer response for version 1

**DOI:** 10.5256/f1000research.55277.r83589

**Published:** 2021-04-22

**Authors:** Nedal Alnawaiseh

**Affiliations:** 1 Department of Public Health, School of Medicine, University of Mutah, Al-Karak, Jordan

This study addressed a critical point, mental health, which is considered a taboo within conservative societies. The authors addressed this issue and clearly clarified its limitation in the manuscript.

 I found it really interesting to cover a spectrum of indicators that assess, or screen, for potential mental health problems among medical students. The question is, then, how could this reflect on the population? Any suggestions?

 Also, medical students are more likely to seek mental health problems if they have access to a clinic. But this is not the norm in conservative societies like Jordan. It may be considered a limitation if the authors did not assess access to mental health professional services. On the same point, how will the screening tools for the positive and negative domains investigated reflect the true problem given that self-reported mental health depression, for example, may be overestimating the actual values. Any thoughts on what the authors expect the true need for mental health advising and counseling among this population? 

 The mental health status of medical students is biased by multiple factors that were not addressed in the study. How will the authors direct future research considering such biases? For example, academic achievement and substance use. 

 On the same page of future research, will next research question encounters worry? Depression? Anxiety? Or what domain? 

 It seems that gender is playing a major role in defining mental health of medical students. Why didn't the authors do a regression analysis to identify the role of gender? This may be due to the fact that the tools used limit further statistical tests? Any other thoughts?

Is the work clearly and accurately presented and does it cite the current literature?

Yes

If applicable, is the statistical analysis and its interpretation appropriate?

Yes

Are all the source data underlying the results available to ensure full reproducibility?

Yes

Is the study design appropriate and is the work technically sound?

Yes

Are the conclusions drawn adequately supported by the results?

Yes

Are sufficient details of methods and analysis provided to allow replication by others?

Yes

Reviewer Expertise:

Epidemiologist, Biostatician, Public health and research expertise.

I confirm that I have read this submission and believe that I have an appropriate level of expertise to confirm that it is of an acceptable scientific standard.

# Response

**Published:** 2021-04-29

**Authors:** khalid kheirallah

**Affiliations:** Jordan University of Science and Technology, Jordan

The authors much appreciate your valuable feedback and thank you for the approval of this manuscript to be considered for publication at F1000Research. Below please note our response in line with your feedback. 

 This study addressed a critical point, mental health, which is considered a taboo within conservative societies. The authors addressed this issue and clearly clarified its limitation in the manuscript.

- **Thank you for this positive feedback and added value of the paper. **

 

 I found it really interesting to cover a spectrum of indicators that assess, or screen, for potential mental health problems among medical students. The question is, then, how could this reflect on the population? Any suggestions?

- **This study addressed such spectrum in order to fine-tune needs of this valuable population. As indicated in our submission, one can simply identify the major mental ill-health concerns as being self-identified among participants by gender and by year level. Top problems could be further explored using future research as indicated in the study. We never aimed to diagnose mental ill-health but rather point to the potential problem among this group. If 10% of self-reported issues are considered high-risk, then this could further identify where interventions could be considered and where the research focus should be. **
- **As for the population, the results reflect an image that reflects on the general population. Our focus then should also consider the general public and other subpopulations.  **

Also, medical students are more likely to seek mental health problems if they have access to a clinic. But this is not the norm in conservative societies like Jordan. It may be considered a limitation if the authors did not assess access to mental health professional services. On the same point, how will the screening tools for the positive and negative domains investigated reflect the true problem given that self-reported mental health depression, for example, may be overestimating the actual values. Any thoughts on what the authors expect the true need for mental health advising and counseling among this population? 

- **It is a major limitation that we did not consider seeking medical help. But this is an issue that was suggested by reviewers of the questionnaire to exclude as it will not be an accurate measure for this taboo-related issue. **
- **As stated earlier, we expect that our screening self-reported results address provide 10% of self-reported issues be an actual estimate then we have an idea about the impact of mental health issues among medical students. **

The mental health status of medical students is biased by multiple factors that were not addressed in the study. How will the authors direct future research considering such biases? For example, academic achievement and substance use. 

- **The top 5 mental health issues identified in the manuscript could be further investigated and explored using fine-tuned questionnaires that consider other potential confounders. This is not added to the manuscript and will be part of the recommendations. **

On the same page of future research, will next research question encounters worry? Depression? Anxiety? Or what domain? 

- **Apparently this research pointed to the top 5 mental health issues (for each gender and year level). These could further be explored using follow-up studies. This was indicated in the study. **

It seems that gender is playing a major role in defining mental health of medical students. Why didn't the authors do a regression analysis to identify the role of gender? This may be due to the fact that the tools used limit further statistical tests? Any other thoughts? 

- **While a regression is a good fit for this paper, we wanted to only shed the light on the major mental health issues and point out to the policy makers of the major ones to be considered in the medical curriculum and medical services provided to students. We did not feel that adding a regression model for a self-reported mental health issue is a good fit as it may not reflect validity of self-reported depression, for example. Future research utilizing standardized screening tools may use advanced statistical analyses and have more variables relevant to mental health. **
